# Fine-scale tracking of wild waterfowl and their impact on highly pathogenic avian influenza outbreaks in the Republic of Korea, 2014–2015

**DOI:** 10.1038/s41598-020-75698-y

**Published:** 2020-10-29

**Authors:** Kyuyoung Lee, Daesung Yu, Beatriz Martínez-López, Hachung Yoon, Sung-Il Kang, Seong-Keun Hong, Ilseob Lee, Yongmyung Kang, Wooseg Jeong, Eunesub Lee

**Affiliations:** 1 Center for Animal Disease Modeling and Surveillance (CADMS), Department of Medicine and Epidemiology, School of Veterinary Medicine, University of California, Davis, CA USA; 2 Veterinary Epidemiology Division, Animal and Plant Quarantine Agency (QIA), Gimcheon, Republic of Korea

**Keywords:** Influenza virus, Animal migration, Animal behaviour, Ecological epidemiology, Viral transmission

## Abstract

Wild migratory waterfowl are considered one of the most important reservoirs and long-distance carriers of highly pathogenic avian influenza (HPAI). Our study aimed to explore the spatial and temporal characteristics of wild migratory waterfowl’s wintering habitat in the Republic of Korea (ROK) and to evaluate the impact of these habitats on the risk of HPAI outbreaks in commercial poultry farms. The habitat use of 344 wild migratory waterfowl over four migration cycles was estimated based on tracking records. The association of habitat use with HPAI H5N8 outbreaks in poultry farms was evaluated using a multilevel logistic regression model. We found that a poultry farm within a wild waterfowl habitat had a 3–8 times higher risk of HPAI outbreak than poultry farms located outside of the habitat. The range of wild waterfowl habitats increased during autumn migration, and was associated with the epidemic peak of HPAI outbreaks on domestic poultry farms in the ROK. Our findings provide a better understanding of the dynamics of HPAI infection in the wildlife–domestic poultry interface and may help to establish early detection, and cost-effective preventive measures.

## Introduction

Highly pathogenic avian influenza (HPAI) virus is an influenza type A virus with high mortality and morbidity in domestic poultry. It is adapted to a broad range of host species from wild birds to humans^1,2^. HPAI infection has been a high-priority concern in the global poultry industry because of the massive financial losses associated with HPAI outbreaks^3–5^. Migratory waterfowl of the genus *Anas*, or “dabbling ducks”, are considered one of the most important reservoirs and long-distance carriers in the global spread of HPAI^1^. This information is supported by experimental evidence of asymptomatic avian influenza virus (AIV) infection and viral shedding in waterfowl^6–12^, the genetic relatedness of AIV strains between poultry and wild birds^13–15^, and the spatial and temporal proximity of HPAI infection in domestic poultry^16–18^.

The poultry industry in the Republic of Korea (ROK) (i.e., South Korea) has suffered each year due to the introduction of novel HPAI strains since the early 2000s^19,20^. In every HPAI outbreak, migration of wild waterfowl from other countries has been suspected as a major source of novel AIV introduction to domestic poultry farms^6,20^. The global outbreak of HPAI H5N8 in 2014 was an unprecedented event that demonstrated the impact of long-distance migration of wild waterfowl on the global spread of this novel HPAI strain^21^. In January 2014, two novel strains of HPAI H5N8 virus were identified in wild waterfowl in the ROK; one of the strains led to HPAI outbreaks in domestic poultry farms in 2014 and 2015^13,17,22^. This HPAI H5N8 strain was subsequently identified in Japan, Europe and North America^23–27^, and was comprised of five gene segments (HA, PB2, PA, M, and NS) from the novel HPAI H5N2 virus in 2014 that caused unprecedented HPAI outbreaks in the US^4,14,28^. The global consortium for H5N8 and related influenza viruses reported phylogenetic, biological and ecological evidence supporting the role of migratory waterfowl as a factor in the long-distance spread of HPAI H5N8 virus^6,29,30^. However, understanding the epidemiological impact of spatial and temporal overlap between domestic poultry farms and wild waterfowl habitats on HPAI outbreaks is still limited. Investigating the introduction of a novel pathogen and its spillover from wildlife to domestic animals has been challenging using conventional epidemiological approaches because of the difficulty in gauging exposure dynamics at the wildlife-domestic interface^31^.

Recent improvements in tracking technology have enabled high-resolution measurement of wildlife habitat use by large and small animals both on land and in the air^32,33^. Traditionally, the identification of wildlife habitat has been limited to a few observations in multiple fixed locations^34,35^. However, this approach has difficulty capturing the spatial and temporal dynamics of wildlife behavior relevant to studying the wildlife-domestic interface. Current light-weight tracking devices using solar-powered batteries can track a small flying animal over several months, recording a high-quality trajectory with limited impact on natural behavior^32,33^. Concurrent with the technological advancement of tracking devices, novel statistical approaches in combination with high computational power allow us to precisely estimate wildlife habitats from collected trajectories^36,37^. The dynamic Brownian bridge movement model (dBBMM) presented by Kranstuaber et al*.*^38^ provides a valuable statistical framework to estimate utilization distribution (UD), a probability density illustrating the relative frequency of an animal's habitat use during the period of observation, derived from a trajectory of the wild animal’s dynamic movement pattern by selecting the best-fit combination of multiple motion variances. Both technical and theoretical advances in trajectory-based habitat estimation give us an opportunity to better explore the complex eco-epidemiology of wildlife^9,39–43^.

Our study focused on defining the spatial and temporal characteristics of the habitats of three representative wild migratory waterfowl species wintering in the ROK. Furthermore, we evaluated the impact of these wild waterfowl’s habitats on the risk of HPAI outbreaks in commercial poultry farm locations. Our results aim to provide a better understanding of the dynamics of wild migratory waterfowl’s habitats at fine spatio-temporal scales, and how these dynamics affects the risk of introduction or transmission of novel pathogens from wildlife to domestic animals. Ultimately, our study presents a novel epidemiological approach combining trajectory-based habitat estimation for monitoring influenza virus at the wildlife-domestic interface and provide a baseline for HPAI risk-based surveillance in commercial poultry farms in the ROK.

## Results

**Figure 1:**
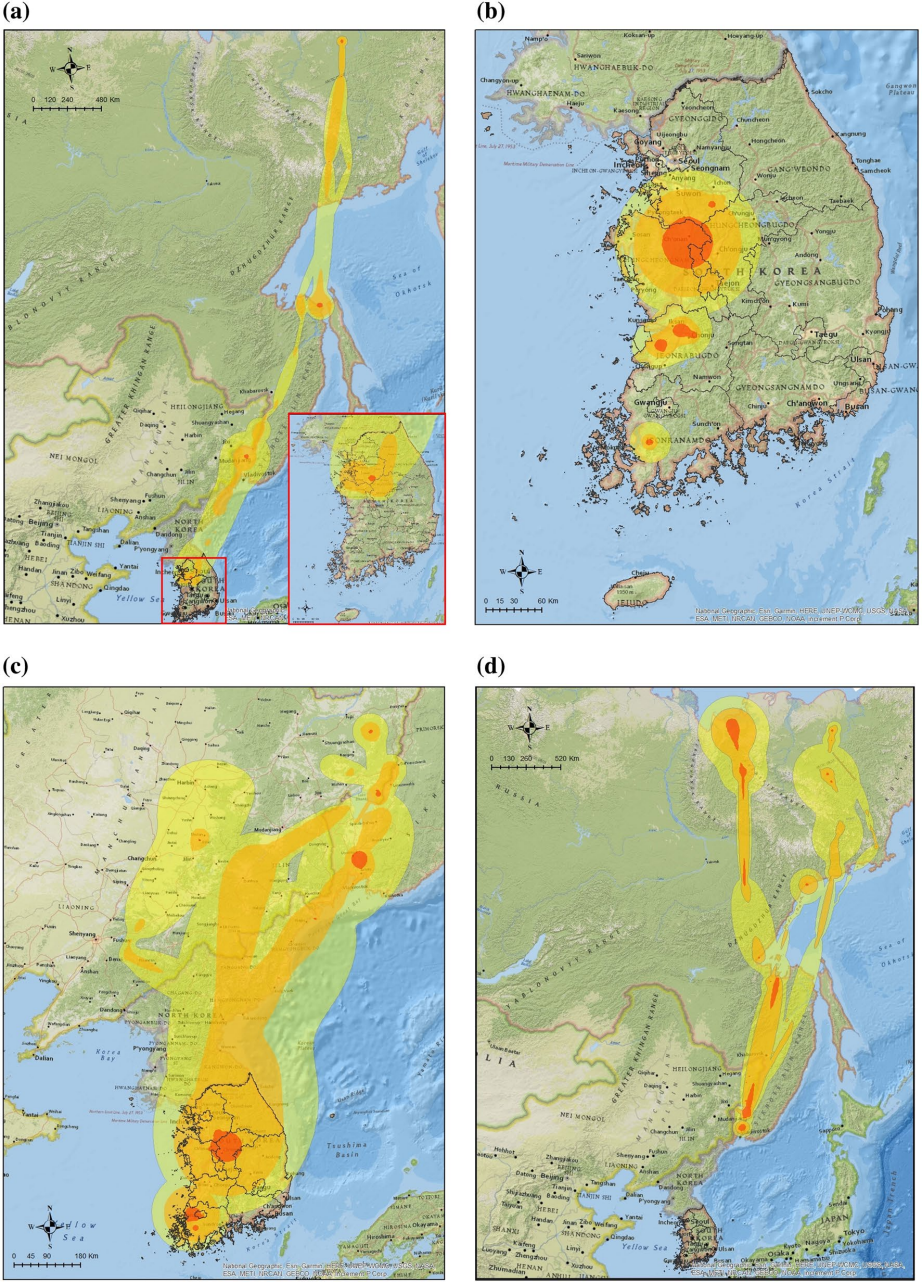

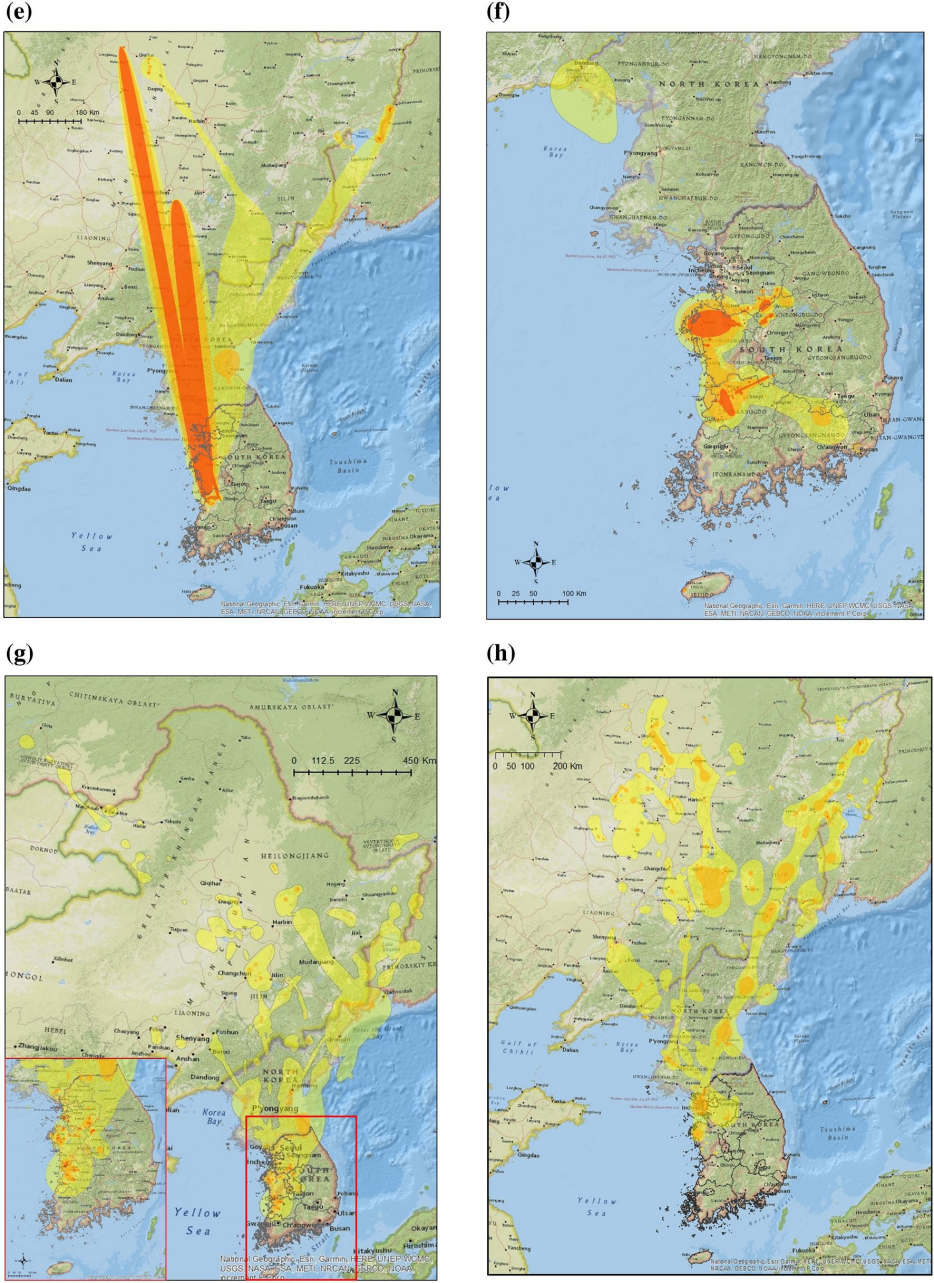

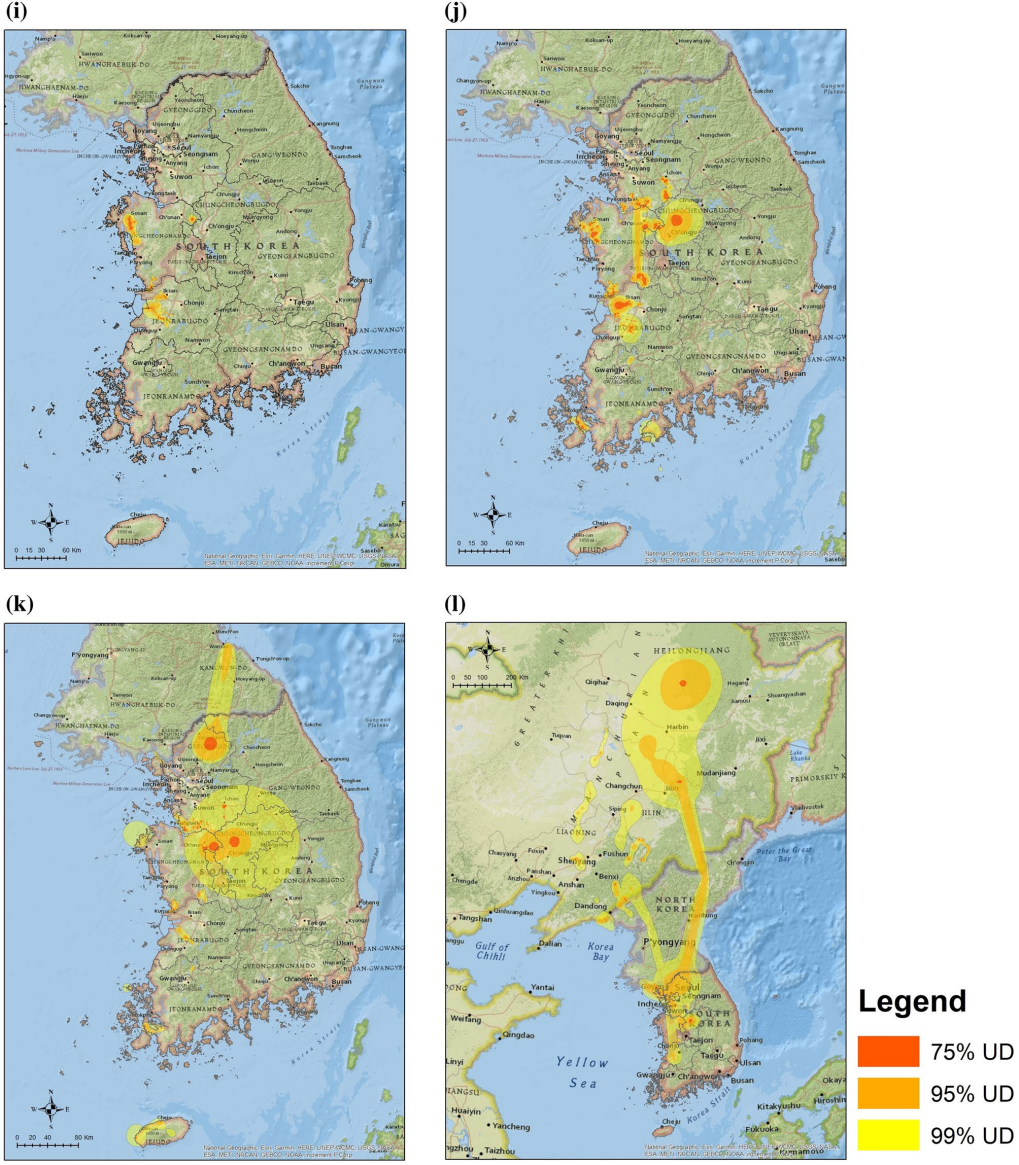
Utilization distributions (UD) of the annual cycle of migration in three species of wild migratory waterfowl wintering in the Republic of Korea from 2013 to 2016. Dark orange, light orange and yellow isopleths indicate 75%, 95%, and 99% cumulative UDs for each waterfowl species in one period, from 2013 to 2016. Common Teals UDs in: (**a**) The autumn migration from September to November (Period, n = 3 birds), (**b**) Wintering from December to January (Period 2, n = 8 birds), (**c**) The spring migration from February to March (Period 3, n = 8 birds), (**d**) Breeding from April to August (Period 4, n = 6 birds). Mallards UDs during: (**e**) Period 1 (n = 65 birds), (**f**) Period 2 (n = 147 birds), (**g**) Period 3 (n = 95 birds), (**h**) Period 4 (n = 41 birds). Spot-billed Ducks UDs during: (**i**) Period 1 (n = 20 bird), (**j**) Period 2 (n = 78 birds), (**k**) Period 3 (n = 44 birds) and (**l**) Period 4 (n = 26 birds). All UDs were estimated and plotted using R version 3.6.1 and ArcMap version 10.7.

Our study estimated the habitat use of 344 wild migratory waterfowl for three major species (Common Teals, Mallards and Spot-billed Ducks) wintering in the ROK from 2013 to 2016 (Table 1). Most migratory waterfowl returned from Northeast China or the far east regions of Russia to western and mid-central provinces of the ROK (JN: Jeollanam-do, JB: Jeollabuk-do, CN: Chungcheongnam-do and CB: Chungcheongbuk-do) for autumn migration between September and November (Fig. 1a,e,i). During the wintering period, from December to January, all three species showed short-range movements within a limited area for feeding or resting; Spot-billed Ducks and Mallards changed their habitats once or twice to southern regions in the western provinces of the ROK (Fig. 1b,f,j). Wild migratory waterfowl expanded their habitat use during the spring migration from February to March. All Common Teals migrated to Northeast China, Primorsky or Khabarovsk regions of Russia (Fig. 1c). 41.1% of the Mallards (39/95) migrated to northeast China, Mongolia, and Russia (Fig. 1g). 2.4% of Spot-billed Duck (1/41) migrated at the end of March; the remaining 97.6% of Spot-billed Ducks (40/41) did not migrate and remained in the ROK (Fig. 1k). During the breeding period from April to August, we observed a secondary migration of Common Teals to the North Chukotka region of Russia (Fig. 1d). 80.5% of Mallards (33/41) migrated to Northern China, Mongolia, or Russia but the remaining 19.5% (8/41) stayed in the ROK (Fig. 1h). 88.5% of Spot-billed Ducks (23/26) did not migrate until April; 11.5% of three birds (3/26) migrated to Northeast China during breeding period (Fig. 1l).

**Table 1:** Summary of wild migratory birds marked and tracked in our study. WT-200, 300 and 500 are the transmitters for mid-large sized waterfowl based on a solar-powered cellular telemetry system [KoEco, The Republic of Korea]. The weight of the device is less than 27 g and it can be used for birds heavier than 700 g (e.g. Mallard). Platform Terminal transmitter (PTT) is the transmitter for small sized waterfowl based on a solar-powered satellite tracking system [Microwave Telemetry, Inc., MD, US]. The weight of device is less than 9.5 g and it can be used for birds lighter than 700 g (e.g. Common Teal).

Species	Year of marking	Tracking device	The number of tracked birds				Tracked birds (success rate)	Marked birds
			Period 1	Period 2	Period 3	Period 4		
Mallard (*Anas platyrhynchos*)	2013	WT-200	8	13	12	0	19 (43.2%)	44
	2014	WT-300	3	34	16	3	48 (56.5%)	85
	2015	WT-500	52	99	48	24	156 (88.6%)	176
	2016	WT-500	2	1	19	14	19 (95.0%)	20
Sub-total			65	147	95	41	242 (74.5%)	325
Common Teal (*Anas crecca*)	2014	PTT	3	3	7	6	7 (50%)	14
	2015	PTT	0	5	1	0	5 (62.5%)	8
Sub-total			3	8	8	6	12 (54.5%)	22
Spot-billed Duck (*Anas poecilorhyncha*)	2013	WT-200	0	0	7	4	7 (41.2%)	17
	2014	WT300 and PTT	6	34	20	11	36 (61.0%)	59
	2015	WT500 and PTT	14	44	17	11	47 (68.1%)	69
Sub-total			20	78	44	26	90 (62.0%)	145
Total			88	233	147	73	344 (69.9%)	492

**Figure 2:**
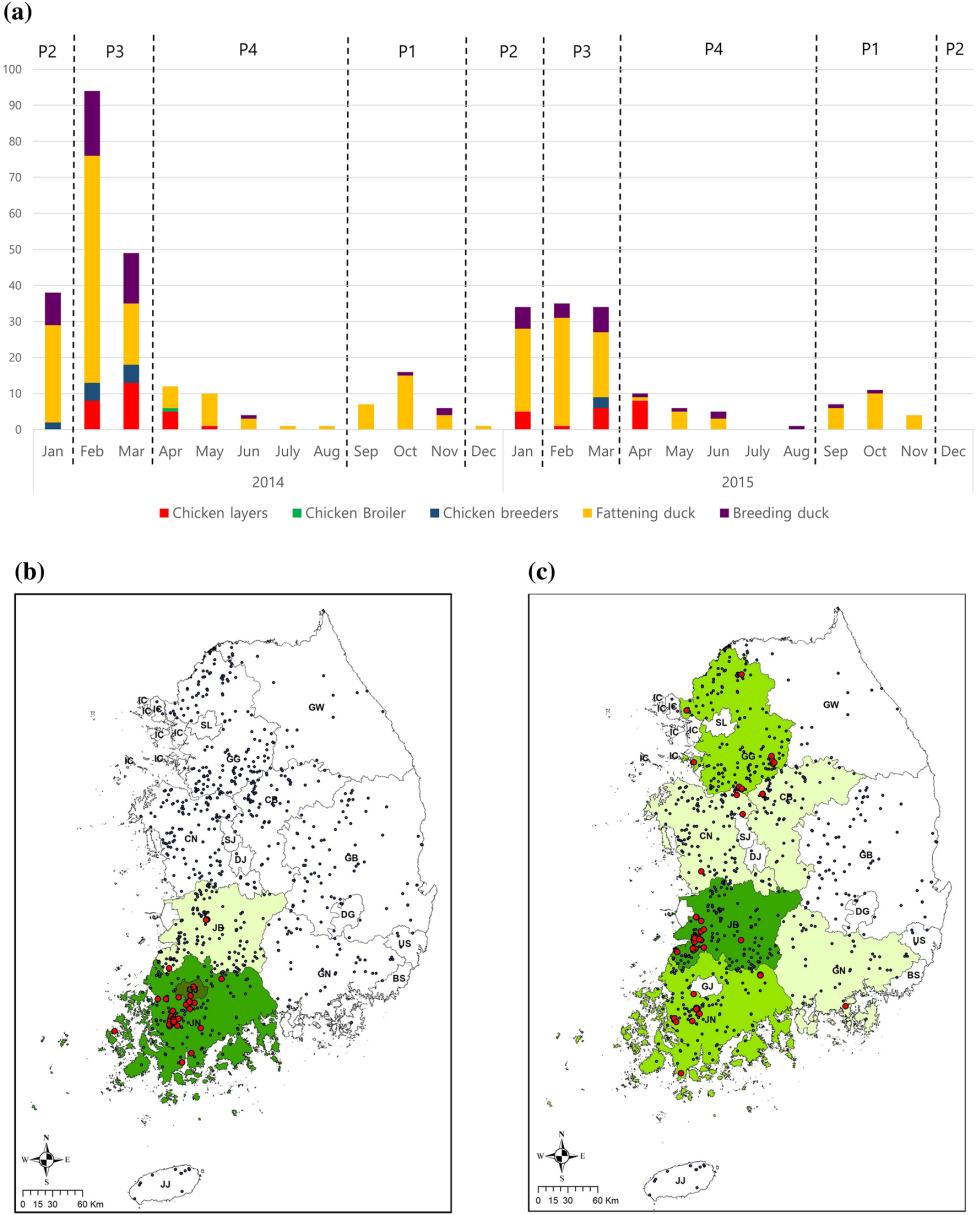

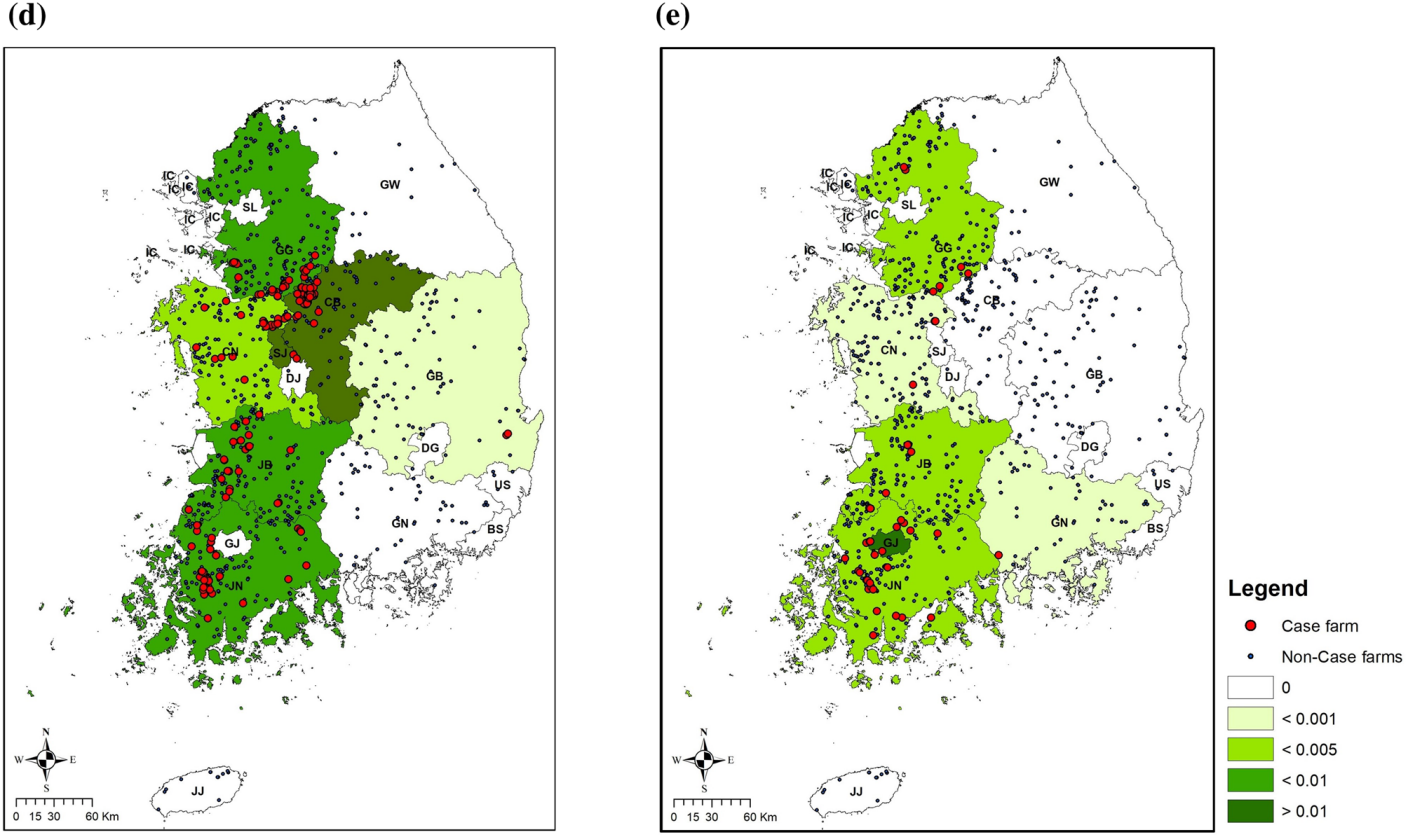
Summary of HPAI H5N8 outbreaks in the Republic of Korea from 2014 to 2016. (**a**) The number of HPAI outbreaks per month in 5 types of commercial poultry farms by the annual cycle of migration of wild migratory waterfowl [Period 1(P1): The autumn migration from September to November, Period 2 (P2): wintering from December to January, Period 3 (P3): the spring migration from February to March and Period 4 (P4): breeding from April to August]. Maps of centroids of HPAI case and non-case farms and incidence of HPAI in each province in (**b**) Period 1, (**c**) Period 2, (**d**) Period 3 and (**e**) Period 4. All Maps were made using ArcMap version 10.7.

A total of 386 HPAI H5N8 outbreaks occurred between Jan. 2014 and Nov. 2015. These were classified by three epidemic waves; 209 HPAI outbreaks in the 1st wave (Jan. 2014 to Aug. 2014), 154 HPAI outbreaks in the 2nd wave (Sep. 2014 to Jun. 2015), and 23 HPAI outbreaks in the 3^rd^ wave (Sep. 2015 to Nov. 2015) (Fig. 2a). The HPAI H5N8 outbreaks occurred on 350 poultry farms; a total of 26 poultry farms were infected more than twice during the three H5N8 epidemics periods. During the three epidemics, duck farms showed a five times higher incidence of H5N8 outbreaks than chicken farms (Fig. 2a). During the autumn migration period, HPAI H5N8 outbreaks only occurred at duck farms in two southwestern provinces (JN and JB) (Fig. 2b). HPAI H5N8 outbreaks were identified in both duck and chicken farms, across all of the western provinces (JN, JB, CN, CB & GG: Gyeonggi-do), and in one southeastern province (GN: Gyeongsangnam-do), during the wintering period (Fig. 2c). The western provinces (JN, JB, CN, CB and GG) showed the highest incidence of infection during the spring migration period compared to the other seasonal periods (Fig. 2d). HPAI H5N8 incidence decreased in these provinces during the breeding period from April to August (Fig. 2e).

**Figure 3:**
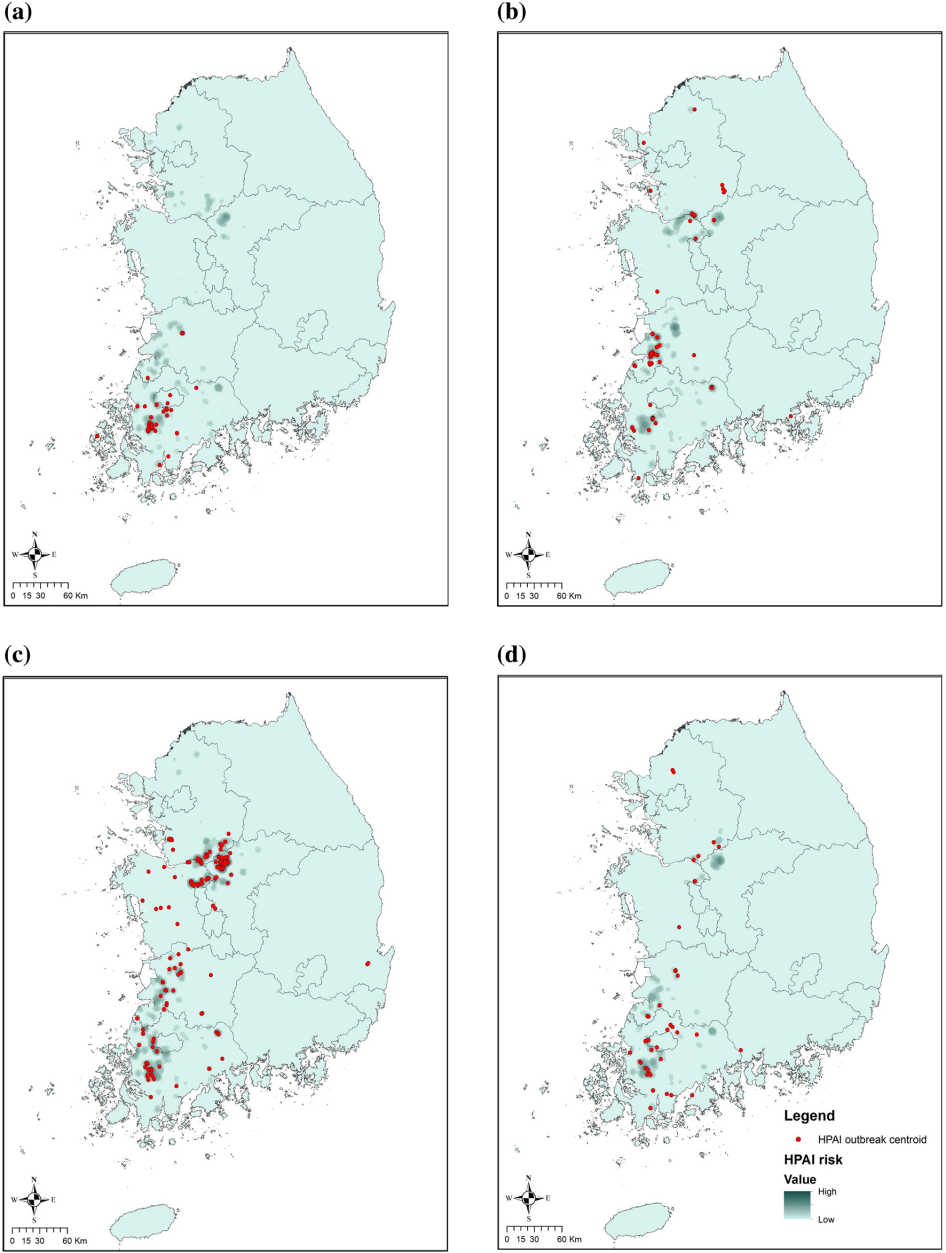

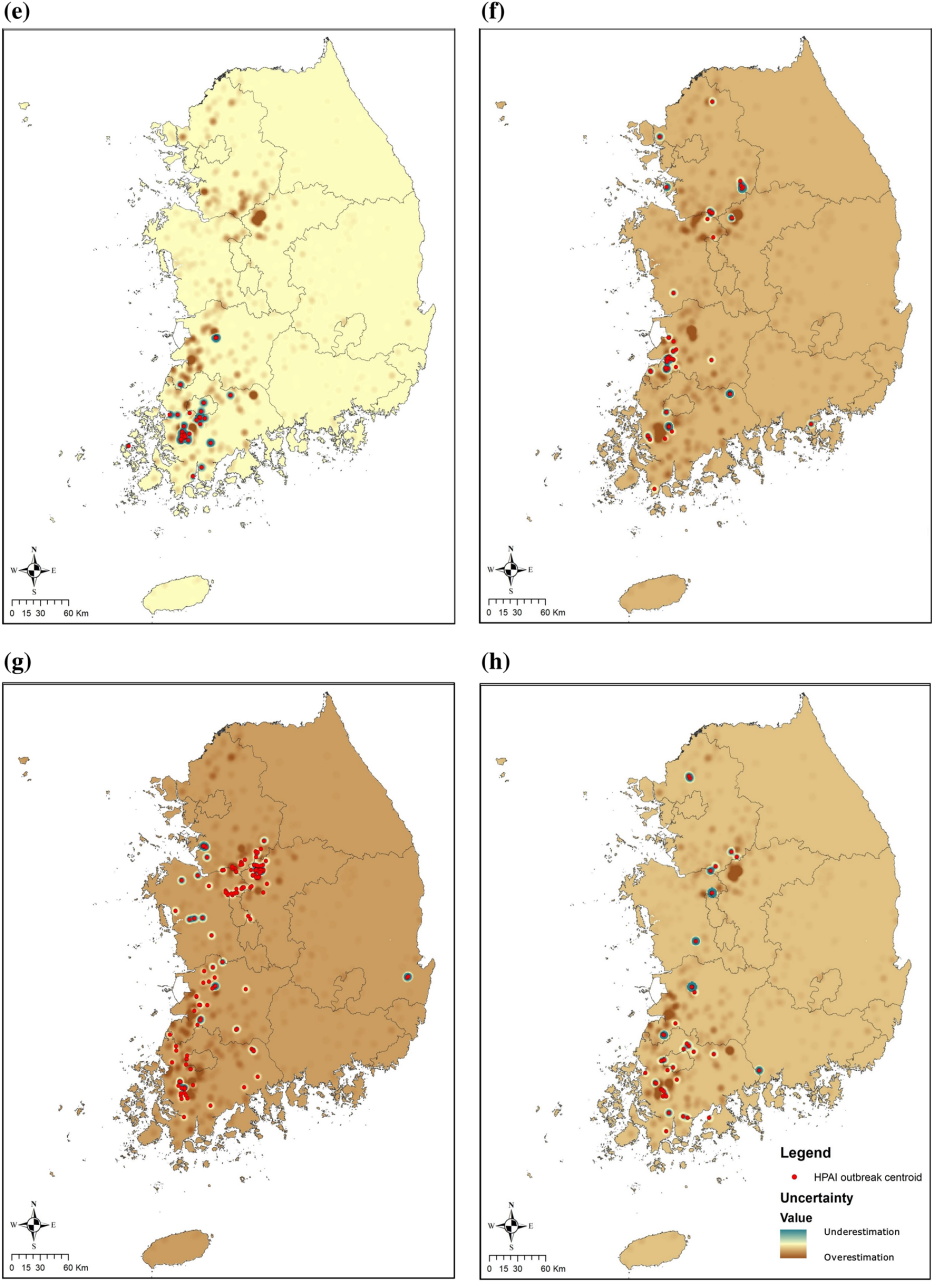
Risk maps and uncertainty maps of HPAI outbreaks in commercial poultry farms predicted by the final model with Kernel density considering migratory waterfowl habitat uses and demographic factors of poultry farms. The risk maps use blue color gradients to visualize the risk of HPAI outbreak in a period from light (low) to dark (high). Risk maps of HPAI outbreaks in commercial poultry farms in (**a**) The autumn migration from September to November (Period 1), (**b**) Wintering from December to January (Period 2), (**c**) The spring migration from February to March (Period 3), and (**d**) Breeding from April to August (Period 4). The uncertainty maps use two-color gradients to visualize the difference between predicted risk and observed risk of HPAI outbreak in a period from brown (overestimation) to green (underestimation). Uncertainty maps of HPAI outbreaks in commercial poultry farms for (**e**) Period 1, (**f**) Period 2, (**g**) Period 3, and (**h**) Period 4. The risk and residuals of HPAI were estimated and plotted using R version 3.6.1 and ArcMap version 10.7.

The habitat predictor selection among three UDs using cumulative probabilities (75, 95 and 99%) determined that the best-fit predictors occurred in the 95% UDs for all three species, except in the best-fit predictor of Common Teals in the period concurrent with an HPAI outbreak (75% UD) (Supplementary Table S1). We found that a commercial poultry farm located within a wild migratory waterfowl habitat during an epidemic had 3–8 times higher odds of HPAI outbreak than poultry farms outside of waterfowl habitat (Table 2). Conversely, poultry farms within the habitats of Mallards and Common Teals during the period *prior* to an HPAI outbreak had 3–4 times lower odds than the farms outside of the habitat (Table 2). Commercial poultry farm type was a risk factor for an HPAI outbreak in all six regression models (Table 2). Duck farms had a significantly higher odds for an HPAI outbreak than chicken farms; breeding duck farms showed the highest odds for an HPAI outbreak among the five types of poultry farms. The flock size of the farm was not a significant predictor in the regression models and was excluded in the final model selection. Our final model included farm type, and geographical location of commercial poultry farms: inside or outside of Common Teals’ and Mallards’ habitats in both the concurrent period, and the period *prior* to an HPAI outbreak, and Spot-billed Ducks’ habitat in the period concurrent with an HPAI outbreak, as five significant predictors (Table 3). Poultry farms within the habitat of all three wild migratory waterfowl in the period concurrent with an HPAI outbreak, showed a positive association with the risk of HPAI outbreak on the poultry farms. On the other hand, poultry farms within the habitat of Mallards and Common Teals in the period *prior* to an HPAI outbreak were negatively associated with the risk of HPAI outbreak. The final model’s ability to predict HPAI outbreaks was high, with an AUC of 0.86 for the ROC curve, and used to generate the HPAI risk map. The predictive HPAI risk map revealed that western and mid-central provinces of ROK had the highest risk for HPAI outbreaks, and that the risk was mostly concentrated during the spring migration period (Fig. 3).

**Table 2:** Results of the multilevel logistic regression model including one variable of wild waterfowl species habitat.

Variable name	OR [95% CI]
**Farm located within the habitat of Common Teals (75% UD) at the period concurrent with an HPAI outbreak **	
No	1
Yes	8.39 [6.36, 11.10]
**Farm located within the habitat of Common Teals (95% UD) at the period prior to an HPAI outbreak**	
No	1
Yes	0.25 [0.19, 0.34]
**Farm located within the habitat of Mallards (95% UD) at the period concurrent with an HPAI outbreak **	
No	1
Yes	3.57 [2.78, 4.59]
**Farm located within the habitat of Mallards (95% UD) at the period prior to an HPAI outbreak**	
No	1
Yes	0.35 [0.25, 0.52]
**Farm located within the habitat of Spot-billed Duck (95% UD) at the period concurrent with an HPAI outbreak**	
No	1
Yes	4.13 [3.03, 5.63]
**Farm located within the habitat of Spot-billed Duck (95% UD) at the period prior to an HPAI outbreak**	
No	1
Yes	3.38 [2.48, 4.60]

**Table 3:** Results of the final multilevel logistic regression model including all variables of wild waterfowl species habitat.

Variable name	OR (95% CI)
**Farm type**	
Chicken breeders	1
Chicken layers	0.92 [0.49, 1.74]
Chicken broilers	0.017 [0.002, 0.13]
Fattening duck	2.99 [1.69, 5.30]
Breeding duck farm	3.85 [2.05, 7.22]
**Farm located within the habitat of Common Teals (75% UD) at the period concurrent with an HPAI outbreak**	
No	1
Yes	4.97 [3.62, 6.82]
**Farm located within the habitat of Common Teals (95% UD) at the period prior to an HPAI outbreak**	
No	1
Yes	0.46 [0.34, 0.61]
**Farm located within the habitat of Mallards (95% UD) at the period concurrent with an HPAI outbreak**	
No	1
Yes	3.55 [2.61, 4.83]
**Farm located within the habitat of Mallards (95% UD) at the period prior to an HPAI outbreak**	
No	1
Yes	0.40 [0.26, 0.60]
**Farm located within the habitat of Spot-billed Duck (95% UD) at the period concurrent with an outbreak**	
No	1
Yes	1.88 [1.28, 2.76]

## Discussion

Our study investigated the spatial characteristics of the annual migration cycles of three wild migratory waterfowl species abundant in the ROK, and their association with the outbreaks of novel HPAI virus on domestic poultry farms, using satellite telemetry data from over 300 birds and national HPAI epidemic data. Our results revealed that commercial poultry farms located within the habitats of Common Teals, Mallards and Spot-billed Ducks, especially within the regions used for resting and feeding via short and mid-range flights (95% UD), showed significantly higher risk for HPAI outbreaks compared to poultry farms outside of the habitat. Consistent with previous studies^6–9,11,12,14,17,19–21,29,44^, our results showed that the core movement of wild migratory waterfowl likely plays an important role in the spillover of HPAI virus between wild birds and domestic poultry farms. Specifically, we found that the expansion of the habitat range of both migratory and non-migratory sub-populations of wild migratory waterfowl during the spring migration coincided with the highest risk of disease outbreak during the HPAI H5N8 epidemics from 2014 to 2015. During spring migration, non-migratory sub-populations of wild waterfowl showed physiological migratory restlessness, called *Zugunruhe*^45^. Although our study did not address the pathway of HPAI virus spillover between wild birds and domestic poultry farms, the increase in short-range movements of non-migratory sub-populations during the spring migration season may result in extensive spillover of HPAI virus between wild birds and domestic poultry farms through direct introduction and/or accidental infection by wild animals residing in proximity to poultry farms^46,47^.

Our findings suggest that commercial duck farms could play a pivotal role in linking the introduction of novel HPAI strains from wild waterfowl and the spread of HPAI outbreaks between poultry farms.

In January 2014, two novel HPAI H5N8 strains were detected in wild waterfowl in a major wintering site of wild migratory waterfowl; one of the novel HPAI strains was subsequently identified in a commercial duck farm close to the detection site^17^. The initial HPAI epidemic wave started at commercial duck farms during the autumn migration period. Duck farms had approximately three times higher risk of HPAI outbreaks than chicken farms during the HPAI epidemics period. Duck farms in ROK have been more susceptible to spillover of novel HPAI strains from wild birds than chicken farms due to the lack of enhanced biosecurity facilities and practices, and the higher density of duck farms around major wintering sites of wild migratory birds compared to chicken farms^40,48–52^. Furthermore, the HPAI H5N8 strain in the ROK is considered to be more host-adapted to the order *Anseriformes* based on its lower mortality among, and higher transmissibility and affinity for, ducks compared to other HPAI strains^30^. Mild clinical signs in ducks could also have led to late detection of HPAI H5N8 infection, potentially allowing “silent” inter-farm transmission to chicken farms as well as additional duck farms. Even though the role of wild migratory waterfowl as carriers of HPAI virus in the transmission chain among poultry farms is still controversial^12,41,53^, our study supports this. Given the epidemiological conditions of the ROK’s poultry industry (Abundance of duck farms), the link between commercial duck farms and wild waterfowl could contribute to amplifying the spillover of novel HPAI strains through the wildlife-domestic interfaces leading to increase inter-farm transmission^48,50,54^. Therefore, risk-based surveillance conducted for both wild waterfowl and duck farms, especially during the autumn migration period, may enable rapid detection of novel HPAI viruses and the initiation of cost-effective preventive measures for HPAI outbreaks in domestic poultry.

Long-distance spring migrations of three wild waterfowl species to breeding grounds in high-latitude regions may play an important role in the international transmission of HPAI virus. The annual census of wild birds in the ROK estimates that more than 300,000 Mallards are annually observed in South Korea. Our tracking records indicated that around 240,000 Mallards (80%) are expected to migrate to breeding sites in Northeast China and Mongolia—the corridor of the East Asia and Australian flyway, and the Central Asian flyways toward the Arctic coast of the Eurasian continent. Although only 11% of marked Spot-billed Ducks migrated to Northeast China, we expect Spot-billed Ducks to have a large number of migratory sub-populations as one of the most abundant wild migratory waterfowl wintering in the ROK (Approximately 1.8 million birds). The spring migration of Mallards and Spot-billed Ducks could potentially contribute to the spread of HPAI strains across the Eurasian continent, considering the significant association between the spatial and temporal overlap of wild migratory waterfowl and poultry farms with HPAI outbreaks, abundance of species, detection of HPAI antigen and antibodies^17^, and the short duration of spring migration (1–2 days) compared to asymptomatic infection duration (3–5 days)^41^. The population of Common Teals is estimated at approximately 30,000 birds in the ROK. All tracked Common Teals migrated to the Bering strait along the East Asia and Australian flyway. The convergence of East Asia and Australian, and Pacific American flyways suggests the exchange of HPAI strains during the post-breeding staging of Common Teals could play an important role in the intercontinental transmission between North America and East Asia^21^. However, the short asymptomatic HPAI infection duration (< 3 days) of the Common teal in comparison to their stopover period (3–5 weeks) makes it unlikely that the Common Teal is a major HPAI carrier throughout their two-stage migration from the ROK to the North Chukotka region of Russia^55^. Investigation of HPAI infection and recombination in the key stopover sites in the Primorsky and Khabarovsk regions of Russia may explain how migration of the Common Teals influences the transmission of AIV between North America and East Asia.

Despite recovering more than 400 satellite tracks of wild migratory waterfowl, with a high tracking success rate (≈ 70%), our study highlights the technical limitations to using tracking devices. According to the annual census of wild birds, more than 250,000 waterfowl belonging to 400 species migrate to winter in ROK. Even though we tracked a much larger number of wild waterfowl compared to previous studies^39,41,43,56^, and observed fine-scale habitat use of the most common and abundant species, our observations are still limited in terms of characterizing the movements of the full diversity of wild waterfowl species that overwinter in the ROK^31^ (400 trajectories ≈ 0.18% of the population). Furthermore, due to the lack of firmly-established evidence defining the HPAI spillover interval between wild birds and domestic poultry, we aggregated the UD of a species not by a regular interval between the habitat use of wild birds and HPAI outbreak in a poultry farm, but by the four periods of the annual migration cycle. Trajectories recorded by different devices posed an additional challenge in aggregating habitat use from individual to species level. The number of tracking points and positional error of the device are key parameters to improve the precision of dBBMM estimation by decreasing the variance of a UD. Across the period of wild bird tracking from 2013 to 2016, our CTT transmitters improved in regard to duration of observation, positional error, and the number of tracking points (from 1 to 12 locations per day) (Table 1). Every year, device advancements improved the precision of individual level UD. However, the aggregation of multiple individual level UDs into a species level UD resulted in a loss of overall precision due to the averaging of variances. Consequently, the aggregation might overestimate the relationship between the wild migratory birds’ habitat use and an HPAI outbreaks by including potential “false positive” case farms that overlapped with the aggregate UD, but were actually spatially and temporally mismatched. In the future, we will attempt to address these limitations by collecting additional tracking records from global data-sharing platforms that provide an accessible web-based database (e.g. Movebank)^57,58^. Furthermore, we will apply of advanced methods to harmonize the aggregation of UDs with inconsistent variance (e.g. weighted linear methods^59^) and/or estimate the suitability of habitat use based on fine-scale environmental factors (e.g. species distribution model)^60–62^.

We acknowledge that the horizontal transmission of HPAI through poultry trade networks between poultry farms was likely a significant factor in its spread, and these events were not fully captured in our study^52^. While the multiple-site production system of the modern livestock industry has significantly improved the efficiency of production, livestock movement between farms is considered an important route of disease transmission^63–69^. Even though the farm type (X_2_), and the flock size of the poultry farm (X_3_) could partially capture the frequency and intensity of poultry shipments, those factors were likely not enough to allow the quantification of the full impact of poultry trade on HPAI outbreaks. In the future, if shipment records between poultry farms and/or the genetic information of the HPAI virus isolated from both wild birds and poultry farms become available, we will explore the impact of inter-farm transmission of HPAI among commercial poultry farms, and evaluate how poultry trade contributes to HPAI outbreaks compared to the role of wild birds^53^.

We provided fine-scale risk maps capturing the intersection of habitat use between wild migratory waterfowl and domestic poultry in different periods, and measured the strength of association between the wildlife-domestic interface and HPAI outbreaks. This approach assists us to have a better epidemiological understanding of the dynamics of the wild-domestic interface in Northeast Asia and its impact on disease spread to commercial livestock farms. Prediction tools for HPAI outbreaks not only inform policy makers in the establishment of cost-effective early detection and preventive measures for novel HPAI strains in the ROK, but also support international initiatives to prevent and control HPAI epidemics globally. The future of global infectious disease surveillance, including HPAI, should follow a One Health framework as many emerging infectious diseases are zoonotic, affecting wildlife, livestock, and humans^31,70–73^. The use of eco-epidemiological approaches, such as the one presented in this study, may assist to evaluate how short, medium, and long-term changes in environmental, climatic, and land-use factors may affect the dynamics of the ecosystem and contribute to disease spread^42,74–76^.

## Methods

### Trajectories of wild migratory waterfowl

Our study collected trajectories of wild migratory waterfowl from the division of veterinary epidemiology in the Animal and Plant Quarantine Agency (APQA) in ROK. A total of 641 wild birds from 15 species were captured and marked from March 2013 to March 2016 in major wintering habitats of migratory waterfowl in the ROK (Supplementary Fig. S1). The trajectories of captured birds were recorded by either Platform Terminal transmitter (PTT-100) using satellite telemetry or Cellular Tracking Technology (CTT), which transmits the signal of a geographical location, with a range error of less than 50 m every 6 h using cellular networks. The CTT transmitter weighed less than 2% of the body weight of the birds. Marked birds were released near their capture location (Table 1). All procedures for marking, capture and handling are reviewed annually and were approved by the research authority of APQA in the ROK.

### Habitat estimation of wild migratory waterfowl

Our study selected a subset of wild migratory waterfowl the trajectories (n = 344) for three species (Common Teals, Mallards and Spot-billed Ducks) from the original 15 species based on three criteria: (1) large population size based on the annual census of wild birds reported by the Ministry of Environment in the ROK, (2) HPAI H5N8 virus or antibody identification during annual wild bird surveillance^17,77,78^ and (3) experimental evidence of asymptomatic HPAI infection^55,79^. Wild migratory waterfowl wintering in the ROK follow an annual cycle of migration at distinct times. To identify the habitat use of three species during the phases of migration, and how this characteristic influences the risk of HPAI outbreak at commercial poultry farms, trajectories were separated into seasons of the annual cycle as defined by the National Institute of Biological Resources in the ROK^16^: Period 1: Autumn migration (September to November); Period 2: Wintering (December to January); Period 3: Spring migration (February to March), and Period 4: Breeding (April to August). Trajectories with less than two locations per a day and shorter than two weeks were excluded (n = 49). Finally, a total of 492 trajectories for 344 birds across four periods were used to estimate the UD, a probability density representing the relative frequency of an animal's occupation in the region during the period of the trajectory observation^36^. For example, 50% of the UD depicts the region where a bird spent 50% of the observation period considering motion variance and positional error. The UD of a trajectory was estimated by dBBMM^38^ using the “Move” package^80^ in R studio (Version 3.6.1)^81,82^. Each UD of a trajectory was summarized by three isopleth maps with cumulative probability contours of 75%, 95% and 99%, which represent the spatial distribution where the bird spent 75%, 95% and 99% of their time during the observation period. Following previous behavioral studies of migratory birds^39,43^, we assumed that the isopleth maps within 75% of the UD were used for resting and core movement during short flights, those between 75 and 95% UD were used for feeding and scouting during mid-range flights, and those between 95 and 99% UD were used for long-distance flights with temporary stops. A bird-level isopleth map was merged with a species-level isopleth map using three cumulative probabilities in four periods with ArcGIS (V 10.7, ESRI, US). Finally, a total of 36 isopleth maps (three species x three probabilities x four periods) were made to identify the most likely habitat use for each of the three species of wild migratory waterfowl for each period.

### Estimation of risk of HPAI outbreaks on poultry farms by overlapping with wild waterfowl habitats

We conducted a cross-sectional study involving HPAI positive case farms and counter-matching samples from nested cohort non-case farms to evaluate the risk of HPAI outbreaks on poultry farms by overlapping with wild waterfowl habitats. During the HPAI H5N8 outbreak period from January 2014 to November 2015, national surveillance was conducted by the Ministry of Agriculture, Food and Rural Affairs, ROK. Every commercial poultry farm registered in the Korean Animal Health Information System (KAHIS) was diagnosed at least once by both HPAI H5N8 antigen and antibody tests. All diagnostic tests were conducted by the animal disease diagnostic laboratory of APQA in the ROK. A “case farm” was defined as a commercial chicken or duck farm with positive test results for either antigen or antibody of HPAI H5N8 virus. A total of 350 commercial poultry farms in 386 HPAI H5N8 outbreaks were included in this study. A “non-case farm” was defined as a commercial chicken or duck farm where neither antigen nor antibodies of HPAI H5N8 were detected through the national surveillance. A total of 772 non-case farms were randomly selected from 37,867 non-case nested cohort farms with 80 (5 × 16) strata to ensure equivalent representation of the five commercial poultry farm types (chicken layers, broilers, chicken breeders, fattening duck, and breeding duck farms) within each of the 16 provinces reported in the poultry industry census published by the Ministry of Agriculture, Food and Rural Affairs, ROK in 2016. The ratio between case and non-case farms (case = 350, non-case = 772; total n = 1,122) equated to a 1:2 ratio to account for the cost of data collection and validation, and to ensure a statistical power higher than 80% (99.999% × 0.91 = 91%). The data were comprised of farm ID, species, production type, flock size, latitude and longitude of the farms’ centroid, radius of the farm, and date of HPAI identification. The data were obtained from KAHIS after data de-identification to preserve the confidentiality of farm owners’ personal information.

A multilevel logistic regression model with random intercepts was used to measure the statistical association between the location of commercial poultry farms inside or outside of estimated habitats of migratory waterfowl, and HPAI outbreaks. Equation (1) was used to estimate the risk of HPAI outbreak on a poultry farm during each period.

1 $$ \begin{aligned} & y_{ijk} \sim Binomial \left( {n_{ijk} ,\pi_{ijk} } \right) \\ & logit \left( {\pi_{ijk} } \right) = \beta_{o} + \beta_{1} X_{1ijk} + \beta_{2} X_{2ijk} + \beta_{3} X_{3ijk} + \beta_{4} X_{4ijk} + \beta_{5} X_{5ijk} \\ & \quad \quad \quad \quad \quad \quad + \beta_{6} X_{6ijk} + \beta_{7} X_{7ijk} + \beta_{8} X_{8ijk} + \beta_{9} X_{9ijk} + \mu_{j} + \mu_{k} \\ \end{aligned} $$

${y}_{ijk}$: The HPAI outbreak in a poultry farm *i* located in province *j* at period *k* (0 = No /1 = Yes).

- Period 1 (*k* = 1): Autumn migration (September to November),
- Period 2 (*k* = 2): Wintering (December to January),
- Period 3 (*k* = 3): Spring migration (February to March),
- Period 4 (*k* = 4): Breeding (April to August).

${\pi }_{ijk}$: The expected probability of HPAI outbreak on a poultry farm *i* located in province *j* at period *k. *${X}_{1ijk}$: The geographical location of the poultry farm *i* inside or outside of Mallards’ isopleth at the migration period *k* concurrent with an HPAI outbreak (0 = Out /1 = In). ${X}_{2ijk}$: The geographical location of the poultry farm *i* inside or outside of Mallards’ isopleth at the migration period *k-1 prior* to an HPAI outbreak (0 = Out /1 = In). ${X}_{3ijk}$: The geographical location of the poultry farm *i* inside or outside of Spot-billed Ducks’ isopleth at the migration period *k* concurrent with an HPAI outbreak (0 = Out /1 = In). ${X}_{4ijk}$: The geographical location of the poultry farm *i* inside or outside of Spot-billed Ducks’ isopleth at the migration period *k-1 prior* to an HPAI outbreak (0 = Out /1 = In). ${X}_{5ijk}$: The geographical location of the poultry farm *i* inside or outside of Common Teals’ isopleth at the migration period *k* concurrent with an HPAI outbreak (0 = Out /1 = In). ${X}_{6ijk}$: The geographical location of the poultry farm *i* inside or outside of Common Teals’ isopleth at the migration period *k-1 prior* to an HPAI outbreak (0 = Out /1 = In). ${X}_{7ijk}$: One of five poultry farm types (Chicken layers, broilers, chicken breeders, fattening duck, and breeding duck farms). ${X}_{8ijk}$: The radius of a poultry farm. ${X}_{9ijk}$: The flock size of a poultry farm. ${\beta }_{o}$: The base intercept of the intercept-varying random effect model. ${\beta }_{1},{\beta }_{2}, \dots {\beta }_{9}$: log odds ratio of individual predictor variables. ${\mu }_{j}, {\mu }_{k}$: The random effects for production site ID of a poultry farm *i* (μ_*k*_) at province j (μ_*i*_).

Our regression model included three binary predictors of whether a poultry farm *i* was located inside or outside of three species’ isopleths (X_1_, X_3_, and X_5_) at the migration period k concurrent with an HPAI outbreak (0 = Out /1 = In). To assess a potential lag from the habitat use of wild waterfowl to HPAI outbreaks in poultry farms, the model included additional three binary predictors of whether a poultry farm *i* was located inside or outside of three species’ isopleths (X_2_, X_4_, and X_6_) at the migration period k − 1 *prior* to an HPAI outbreak (0 = Out /1 = In). Our study selected one predictor among 75%, 95%, and 99% of cumulative probability isopleths for the location of the poultry farm in each of three species’ habitat (X_1_ to X_6_). Goodness of fits of candidate regression models including each UD isopleth predictor were compared using Akaike information criterion (AIC) and Area Under the Curve (AUC) of the Receiver Operating Characteristics (ROC) curve (Table S1). One of five poultry farm types (X_7_), the radius of a poultry farm (X_8_), the flock size of a poultry farm (X_9_) and their interactions were included as covariates in the model. Our model included two random effects, one for the production site ID of a poultry farm (μ_*k*_) to account for the repeated measures design (i.e., four observations of HPAI within each farm, one for each period of the annual cycle of migration), and another for the province of a farm (μ_*i*_) to account for spatial clustering of farms within the same province. The final model was built using backward variable selection and a final model was selected based on the lowest AIC. The final model was used to create risk prediction maps for an HPAI outbreaks in the ROK across four periods, by simple spatial smoothing methods (i.e. Kernel density). We also created uncertainty maps to present the variation of predicted probability within the risk maps. The predicted probability and Pearson residual of an HPAI outbreak around a poultry farm were estimated using Kernel density with the weight of 6 km bandwidth in ArcGIS (V 10.7, ESRI, US). All statistical analyses were performed in R studio (V 1.1.463)^81,82^. All methods were conducted in accordance with the relevant guidelines and regulations of the research authority of APQA in the ROK.

## Supplementary information

Supplementary Information
