# Supplementary material for: Fine-scale tracking of wild waterfowl and their impact on highly pathogenic avian influenza outbreaks in the Republic of Korea, 2014–2015
Source: Sci Rep. 2020 Oct 29;10:18631. doi: 10.1038/s41598-020-75698-y (PMC7596240; doi:10.1038/s41598-020-75698-y)
Supplement: Supplementary file 1 — Supplementary Information [file 41598_2020_75698_MOESM1_ESM.docx]

**
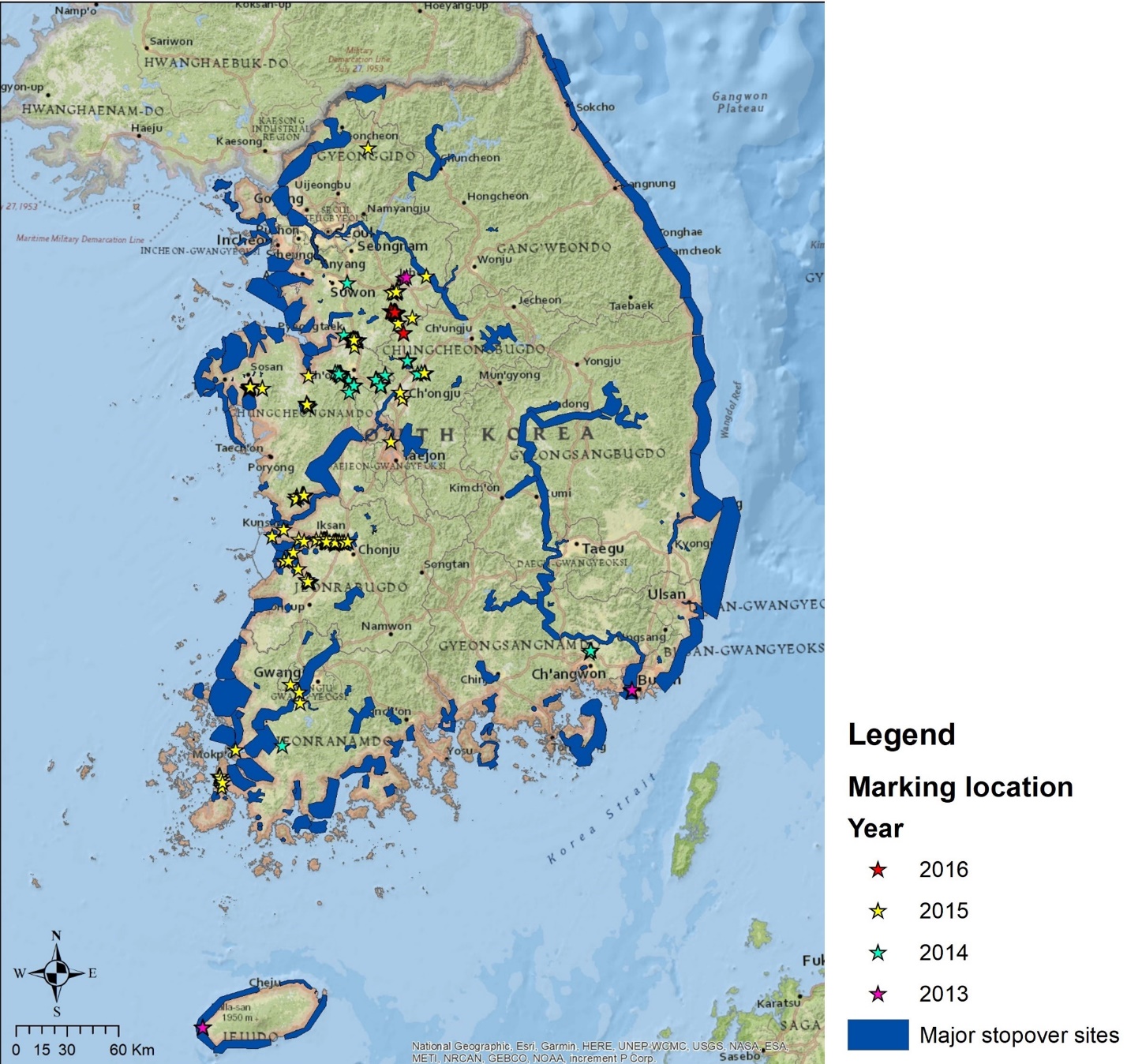
**

**Supplementary Figure 1. Major stopover sites of wild migratory waterfowl in the Republic of Korea and centroids of marking locations from 2013-2016.** The map was plotted using ArcMap version 10.7

**Supplementary Table 1. Results of selection of migratory birds’ habitat predictors among three different UDs of each species (75%, 95% and 99%)**

| Model predictors | Odds ratio | p-value | AIC | AUC | Selection |
| --- | --- | --- | --- | --- | --- |
| Farm located within the habitat of Common Teals (75% UD) at the period concurrent with an HPAI outbreak | 8.39 [6.36, 11.10] | <0.001 | 2068.3 | 0.8423 | Yes |
| Farm located within the habitat of Common Teals (95% UD) at the period concurrent with an HPAI outbreak | 2.08 [1.64, 2.64] | <0.001 | 2251.6 | 0.79 | No |
| Farm located within the habitat of Common Teals (99% UD) at the period concurrent with an HPAI outbreak | 2.54 [1.99, 3.24] | <0.001 | 2229.1 | 0.7977 | No |
| Farm located within the habitat of Spot-billed Duck (75% UD) at the period concurrent with an HPAI outbreak | 2.97 [1.52, 5.80] | <0.001 | 2279.7 | 0.7932 | No |
| Farm located within the habitat of Spot-billed Duck (95% UD) at the period concurrent with an HPAI outbreak | 4.13 [3.03, 5.63] | <0.001 | 2209.7 | 0.8086 | Yes |
| Farm located within the habitat of Spot-billed Duck (99% UD) at the period concurrent with an HPAI outbreak | 2.20 [1.67, 2.88] | <0.001 | 2256.9 | 0.81 | No |
| Farm located within the habitat of Mallards (75% UD) at the period concurrent with an HPAI outbreak | 2.97 [1.99, 4.44] | <0.001 | 2263.3 | 0.7785 | No |
| Farm located within the habitat of Mallards (95% UD) at the period concurrent with an HPAI outbreak | 3.57 [2.78, 4.59] | <0.001 | 2194 | 0.8118 | Yes |
| Farm located within the habitat of Mallards (99% UD) at the period concurrent with an HPAI outbreak | 2.67 [2.08, 3.43] | <0.001 | 2228.7 | 0.8013 | No |
| Farm located within the habitat of Common Teals (75% UD) at the period prior to an HPAI outbreak | 0.18 [0.10, 0.32] | <0.001 | 2232.2 | 0.8034 | No |
| Farm located within the habitat of Common Teals (95% UD) at the period prior to an HPAI outbreak | 0.25 [0.19, 0.34] | <0.001 | 2177.7 | 0.8176 | Yes |
| Farm located within the habitat of Common Teals (99% UD) at the period prior to an HPAI outbreak | 0.28 [0.22, 0.36] | <0.001 | 2178.2 | 0.8143 | No |
| Farm located within the habitat of Spot-billed Duck (75% UD) at the period prior to an HPAI outbreak | 0.67 [0.23, 1.86] | 0.43 | 2289.3 | 0.7779 | No |
| Farm located within the habitat of Spot-billed Duck (95% UD) at the period prior to an HPAI outbreak | 3.38 [2.48, 4.60] | <0.001 | 2233 | 0.818 | Yes |
| Farm located within the habitat of Spot-billed Duck (99% UD) at the period prior to an HPAI outbreak | 2.72 [2.07, 3.57] | <0.001 | 2234.3 | 0.8154 | No |
| Farm located within the habitat of Mallards (75% UD) at the period prior to an HPAI outbreak | 0.17 [0.06, 0.47] | <0.001 | 2269.5 | 0.7847 | No |
| Farm located within the habitat of Mallards (95% UD) at the period prior to an HPAI outbreak | 0.35 [0.25, 0.52] | <0.001 | 2251.3 | 0.8002 | Yes |
| Farm located within the habitat of Mallards (99% UD) at the period prior to an HPAI outbreak | 1.31 [1.02, 1.68] | 0.034 | 2283.7 | 0.7929 | No |
